# Assessment of Hypofluorescent Foci on Late-Phase Indocyanine Green Angiography in Central Serous Chorioretinopathy

**DOI:** 10.3390/jcm10102178

**Published:** 2021-05-18

**Authors:** Ari Shinojima, Yoko Ozawa, Atsuro Uchida, Norihiro Nagai, Hajime Shinoda, Toshihide Kurihara, Misa Suzuki, Sakiko Minami, Kazuno Negishi, Kazuo Tsubota

**Affiliations:** 1 Department of Ophthalmology, Keio University School of Medicine, Tokyo 160-8582, Japan; uchidats@gmail.com (A.U.); nagai@a5.keio.jp (N.N.); shinoha@mac.com (H.S.); kurihara@z8.keio.jp (T.K.); misa.suzuki@suzukiganka.page (M.S.); saki.love5@icloud.com (S.M.); kazunonegishi@keio.jp (K.N.); tsubota@z3.keio.jp (K.T.); 2 Department of Ophthalmology, St. Luke’s International University, 10-1 Akashi-cho, Chuo-ku, Tokyo 104-0044, Japan; 3 Department of Ophthalmology, St. Luke’s International Hospital, 9-1 Akashi-cho, Chuo-ku, Tokyo 104-8560, Japan; 4 Department of Ophthalmology, Yokohama City University, 3-9 Fukuura, Kanazawa-ku, Yokohama 236-0004, Japan

**Keywords:** autofluorescence, axial length, choroidal thickness, fluorescein angiography, imaging, indocyanine green angiography, near-infrared, retinal thickness

## Abstract

To assess the hypofluorescent foci (HFF) on late-phase indocyanine green angiography (ICGA) in central serous chorioretinopathy (CSC) using short-wavelength fundus autofluorescence (SW-FAF), near-infrared autofluorescence (NIR-AF), and fluorescein angiography (FA). The HFF area on late-phase ICGA for at least 20 min was compared with the area of abnormal foci on SW-FAF, NIR-AF, and FA. In 14 consecutive patients (12 men, including 1 with bilateral CSC; and 2 women with unilateral CSC), four kinds of images of 27 eyes were acquired. The mean age ± standard deviation (range) was 46 ± 9.2 years (31–69 years). The HFF on late-phase ICGA were found in 23 eyes (in all 15 CSC eyes and the contralateral 8 eyes). From the results of simple regression analysis, we obtained the following three formulas. The HFF area on ICGA = 1.058 × [abnormal SW-FAF area] + 0.135, the HFF area on ICGA = 1.001 × [abnormal NIR-AF area] + 0.015, and the HFF area on ICGA = 1.089 × [abnormal FA area] + 0.135. Compared to SW-FAF and FA, NIR-AF was found to be the easiest method to detect the HFF on late-phase ICGA, which may indicate melanin abnormalities, especially a decrease, in the retinal pigment epithelium.

## 1. Introduction

Central serous chorioretinopathy (CSC) is a disease that most commonly occurs in men (9.9/100,000), mainly in their 30s to 50s, and its incidence is six times higher than that in women (1.7/100,000). It causes localized serous retinal detachment (SRD) in the macula [1]. In 2009, a study using enhanced depth imaging (EDI) of spectral-domain optical coherence tomography (OCT) showed that the choroidal thickness in CSC is significantly greater than that in normal eyes [2]. Even in a large number of sample sizes, subfoveal choroidal thickness is significantly greater than that in healthy eyes in both acute and chronic CSC [3]. We have previously reported that all eyes with CSC and two-thirds of the fellow eyes showed hypofluorescent foci (HFF) on late-phase indocyanine green angiography (ICGA) over 20 min [4,5]. Late-phase ICGA also revealed significantly greater choroidal thickness in eyes with HFF than in eyes without HFF among patients with CSC [4]. We clarified that these HFF significantly corresponded to abnormal hyperreflective lesions extending from Bruch’s membrane to the choriocapillaris level on enface-OCT images [4], and that these were a significant risk factor for an SRD [5]. Pauleikhoff et al. reported that lipids, such as neutral lipids and phospholipids, accumulated irregularly in Bruch’s membrane with aging [6]. Moore et al. also reported that lipids accumulated in the macular Bruch’s membrane-choroid complex with aging, and hydraulic conductivity was below 50% over 25 years old and below 20% over 30 years old [7]. Considering the age of onset of CSC, CSC may be associated with aging and lipid accumulation in Bruch’s membrane.

The retinal pigment epithelium (RPE) contains melanin, melanolipofuscin, and lipofuscin [8]. Studies have suggested that the short-wavelength fundus autofluorescence (SW-FAF) signal originates from lipofuscin and melanolipofuscin, whereas the long-wavelength near-infrared autofluorescence (NIR-AF) signal originates from melanin and melanolipofuscin [9,10,11]. Studies have also provided consistent evidence that the distribution of NIR-AF corresponds to RPE melanin distribution [12,13]. Thus, the two autofluorescence modalities provide complementary information about the RPE in retinal pathologies.

It is reported that fluorescence in the RPE over the drusen is less intense than that of the adjacent RPE on ICGA [14]. Chang et al. cultured primary human RPE cells incubated with ICG in vitro [15], and they found that ICG was taken up by normal RPE, but decreased in ouabain-damaged RPE. Local accumulation of lipids below the RPE or other aging-related metabolic changes may damage the RPE itself and disturb the intake of ICG, which may result in the appearance of HFF on late-phase ICGA and on NIR-AF due to decreased levels of pigments, such as melanin and melanolipofuscin [16]. Changes in fluorescein angiography (FA), such as window defects, pooling, leakage, and staining, are also related to the RPE changes in CSC.

The appearance of HFF on late-phase ICGA has the potential to show changes in pathology in vivo, but the testing is laborious and invasive. The changes around the RPE should be visible in other modalities. In this study, we comprehensively assessed the HFF on late-phase ICGA imaging by using SW-FAF, NIR-AF, and FA.

## 2. Materials and Methods

This study was conducted at the Department of Ophthalmology, Keio University Hospital, Tokyo, Japan, between October 2019 and March 2020. Data of all consecutive patients with CSC who underwent consultation during this period were prospectively assessed. All procedures adhered to the tenets of the Declaration of Helsinki. Written informed consent was obtained from all patients before they underwent angiography and other examinations. This study was approved by the Keio University School of Medicine Ethics Committee (Approval number 20190085/UMIN000038063).

The eligibility criterion was CSC in either eye. The exclusion criteria were as follows: patients with drusen within 30 degrees, drusenoid or hemorrhagic pigment epithelium detachment, high myopia (<−6.0 diopters), dome-shaped macula, vitreomacular traction syndrome, eyes with choroidal neovascularization detected by OCT angiography, and any history of uveitis or other retinal diseases.

We obtained images using SPECTRALIS HRA+OCT2; Heidelberg Engineering, Heidelberg, Germany. Images created by using SW-FAF, NIR-AF, FA, and ICGA for at least 20 min were analyzed. SW-FAF images were acquired at an excitation wavelength of 488 nm and an emission spectrum over 500 nm, and NIR-AF images were acquired at an excitation wavelength of 787 nm and an emission spectrum over 820 nm [13].

For the data processing, we used Microsoft PowerPoint 2019 (version 2102) and ImageJ (version 1.52a), which is an open-source image processing and analysis program written in Java by Wayne Rasband, National Institutes of Health, Bethesda, MD, USA. 

In 14 consecutive patients (12 men, including 1 with bilateral CSC; and 2 women with unilateral CSC), four kinds of images (SW-FAF, NIR-AF, FA, and ICGA) of 28 eyes were acquired. Images of each eye were obtained on the same day. The HFF on late-phase ICGA were defined as clearly hypofluorescent compared to iso-fluorescent on late-phase ICGA. In chronic CSC, HFF may sometimes include hyperfluorescent foci, which was also defined as HFF. On the PowerPoint, we drew areas containing HFF on late-phase ICGA images using the ‘Curve’, and we then colored those HFF areas. If there were many abnormal HFF, the top biggest 3 abnormal foci on late-phase ICGA were measured. To investigate how many HFF obtained on late-ICGA could be picked up by the other three types of images (SW-FAF, NIR-AF, and FA), we similarly selected abnormal foci that matched the HFF of the ICGA. After confirming that all image ratios matched, we saved the image under another file name.

We then used ImageJ and selected the ‘Wand (tracing) tool’, ‘Analyze’, and ‘Measure’. Since abnormal area sizes were shown on the screen, we converted the real size from the ratio of each image. We repeated the same steps for processing each image, and the HFF areas on late-phase ICGA were compared with the area of abnormal foci on SW-FAF, NIR-AF, and FA images, which corresponded to the HFF on late-phase ICGA images.

Simple regression analyses were performed using IBM SPSS Statistics for Windows, Version 27.0 (IBM Corp., Armonk, NY, USA).

## 3. Results

In 14 consecutive patients (12 men, including 1 with bilateral CSC; and 2 women with only unilateral CSC), 28 eyes were analyzed. The mean age ± standard deviation (range) was 46 ± 9.2 years (31–69 years), and the mean symptom duration was 30.0 ± 72.9 months (0.5–288 months). The mean subfoveal choroidal thickness was 417.2 ± 103.5 µm (265–624 µm), and the mean axial length was 24.1 ± 1.0 mm (22.3–25.9 mm). Only one patient had a history of prior/ongoing corticosteroid use.

There were four patients with unilateral CSC, where the contralateral unaffected eye had no HFF on late-phase ICGA. In addition, one eye showed quiescent choroidal neovascularization using OCT angiography in the contralateral eye of CSC. Therefore, we excluded five eyes from the HFF analysis. The mean HFF area size on late-phase ICGA over 20 min was 3.9 ± 6.9 mm^2^ (0.03–26.5 mm^2^) (*n* = 23). The mean abnormal foci area on SW-FAF was 3.5 ± 6.5 mm^2^ (0.03–26.4 mm^2^) (*n* = 23). The mean abnormal foci area on NIR-AF was 3.8 ± 6.9 mm^2^ (0.03–26.5 mm^2^) (*n* = 23). The mean abnormal foci area on FA was 3.4 ± 6.2 mm^2^ (0.01–26.3 mm^2^) (*n* = 23).

**Figure 1:**
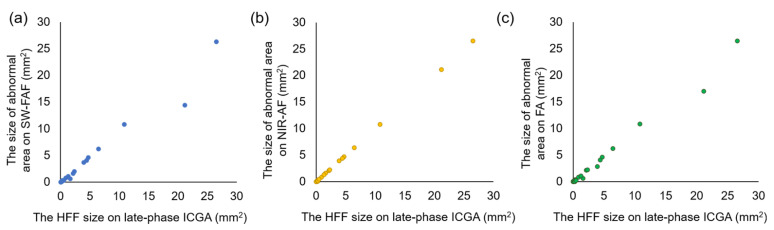
(**a**) Scatter plot of the HFF size of late-phase ICGA (mm^2^) and the size of abnormal area on SW-FAF (mm^2^), (**b**) the scatter plot of the HFF size of late-phase ICGA (mm^2^) and the size of abnormal area on NIR-AF (mm^2^), and (**c**) the scatter plot of the HFF size of late-phase ICGA (mm^2^) and the size of abnormal area on FA (mm^2^).

Figure 1 shows the scatter plot of the HFF size of late-phase ICGA (mm^2^) and the size of abnormal area on SW-FAF (mm^2^), the size of abnormal area on FA (mm^2^), and the size of abnormal area on NIR-AF (mm^2^) (Figure 1). 

From the results of simple regression analysis, we obtained the following three formulas. The HFF area on ICGA = 1.058 × [abnormal SW-FAF area] + 0.135, the HFF area on ICGA = 1.001 × [abnormal NIR-AF area] + 0.015, and the HFF area on ICGA = 1.089 × [abnormal FA area] + 0.135. Compared to SW-FAF and FA, NIR-AF was found to be the easiest method to detect the HFF found in the late-phase of ICGA.

**Figure 2:**
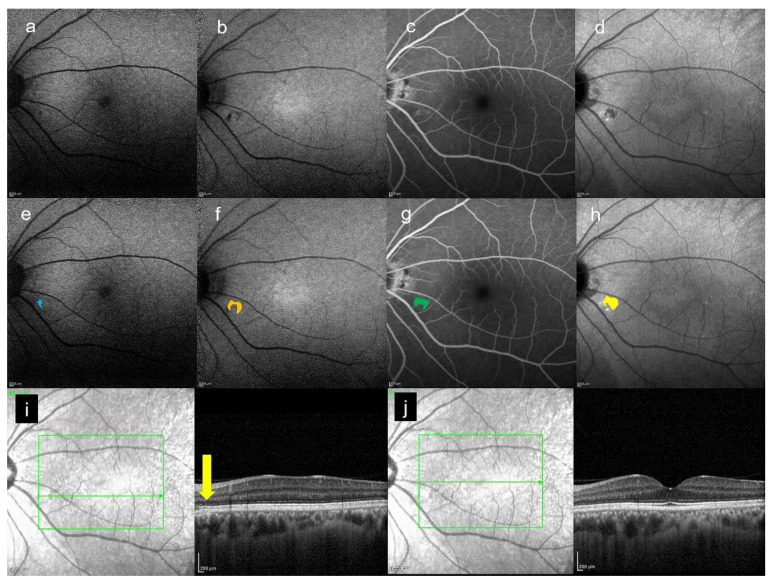
A 39-year-old man. All images are from the contralateral eye of CSC eye. (**a**) SW-FAF image, (**b**) NIR-AF image, (**c**) FA image acquired at 19 min, and (**d**) ICGA image acquired at 27 min. All images were acquired on the same day. (**e**) Abnormal foci which were colored in blue on (**a**), which are within the HFF area on (**d**). The area is 0.26 mm^2^. (**f**) Abnormal foci which were colored in orange on (**b**), which are within the HFF area on (**d**). The area is 0.51 mm^2^. (**g**) Abnormal foci which were colored in green on (**c**), which are within the HFF on (**d**). The area is 0.37 mm^2^. (**h**) The HFF which were colored in yellow on (**d**). The area is 0.51 mm^2^. (**i**) Infrared + OCT (horizontal section) through the abnormal foci (yellow arrow), (**j**) Infrared + OCT (horizontal section) through the fovea.

**Figure 3:**
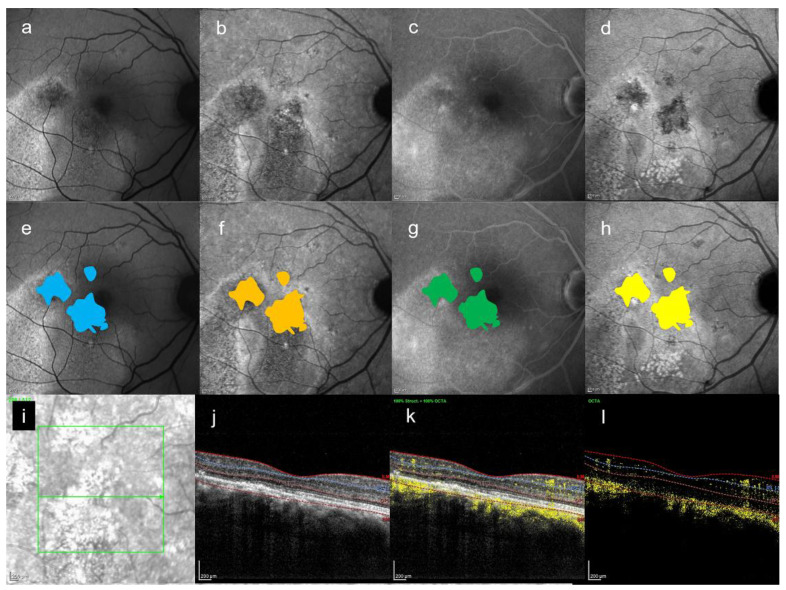
A 48-year-old man with bilateral CSC. (**a**) SW-FAF image, (**b**) NIR-AF image, (**c**) FA image acquired at 19 min, and (**d**) ICGA image acquired at 27 min. All images were acquired on the same day. (**e**) Abnormal foci which were colored in blue on (**a**), which are within the HFF area on (**d**). The area is 4.1 mm^2^. (**f**) Abnormal foci which were colored in orange on (**b**), which are within the HFF area on (d). The area is 4.4 mm^2^. (**g**) Abnormal foci which were colored in green on (**c**), which are within the HFF on (**d**). The area is 4.1 mm^2^. (**h**) The HFF which were colored in yellow on (**d**). The area is 4.4 mm^2^. (**i**,**j**) Infrared + OCT (horizontal section) through the fovea. (**i**,**k**) Infrared + OCT angiography (horizontal section) through the fovea. (**i**,**l**) The image of (**k**–**j**). There is no choroidal neovascularization.

**Figure 4:**
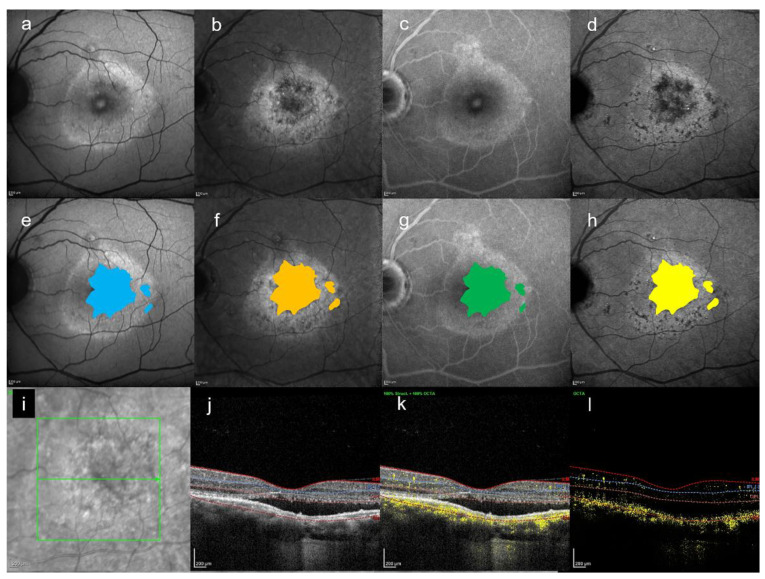
A 48-year-old man with bilateral CSC. All images are the fellow eye of the patient described in Figure 3. (**a**) SW-FAF image, (**b**) NIR-AF image, (**c**) FA image acquired at 19 min, and (**d**) ICGA image acquired at 26 min. All images were acquired on the same day. (**e**) Abnormal foci which were colored in blue on (**a**), which are within the HFF area on (**d**). The area is 4.6 mm^2^. (**f**) Abnormal foci which were colored in orange on (**b**), which are within the HFF area on (**d**). The area is 4.7 mm^2^. (**g**) Abnormal foci which were colored in green on (**c**), which are within the HFF on (**d**). The area is 4.6 mm^2^. (**h**) The HFF which were colored in yellow on (**d**). The area is 4.7 mm^2^. (**i**,**j**) Infrared + OCT (horizontal section) through the fovea. (**i**,**k**) Infrared + OCT angiography (horizontal section) through the fovea. (**i**,**l**) The image of (**k**–**j**). There is no choroidal neovascularization but a focal choroidal excavation.

Figure 2, Figure 3 and Figure 4 are the representative images of this study. Each image by multimodal imaging shows various findings.

## 4. Discussion

In this study, we could detect any abnormal foci on SW-FAF, NIR-AF, and FA within the HFF area on late-phase ICGA. In particular, it was found that NIR-AF easily detected the HFF seen in the late-ICGA visually (Figure 2, Figure 3 and Figure 4). Although ICGA and NIR-AF images are obtained by long wavelength, the findings seen on two modalities were slightly different by using ICG. In this small case series, any HFF differences on late-ICGA were found in both chronic and acute CSC. In acute CSC, simple HFF on late-ICGA were the most common. On the other hand, in chronic CSC, mosaic HFF (a mixture of hyper- and hypofluorescence) were seen. The area of HFF may correspond to atrophy of the RPE, and hyperfluorescent foci may correspond to hyperplasia of the RPE.

In ICGA, we can observe the course of the dye of ICG changing in vivo from the early phase to the late phase. It is reported that the fluorescence of the RPE over the surface of drusen in the eye of a monkey by using ICG was less intense than that of the adjacent RPE [14]. Drawing from the experiment of cultured primary human RPE cells incubated with ICG in vitro, the uptake of ICG was affected by the level of damage of the RPE. Normal RPE cells can uptake ICG almost 100%, but the RPE cell groups with 24 h ouabain-induced damage could uptake ICG only about 15%. On the other hand, the paradoxical increased uptake of ICG into the RPE cells after more prolonged 72 h exposure to ouabain can cause about 90%, which is thought to be due to ICG’s diffusion through the damaged cell membrane [15]. Not limited to drusen [14], accumulated lipids at the Bruch’s membrane level may also make abnormal RPE cells incapable of ICG uptake and can cause them to remain hypofluorescent, in contrast to the surrounding healthy RPE on late-phase ICGA [5,15].

The NIR-AF signal is thought to originate from melanin and melanolipofuscin [9,10,11]. Focal dysfunction of a cluster of RPE cells may cause abnormal melanin production or the loss of melanin granules [5], which may result in the HFF on the NIR-AF image, suggesting the reduction in melanin-related substances in the RPE. Such damaged RPE cells may have difficulties in uptake of ICG, according to the reports by Chang et al. [15]. 

Although SW-FAF and FA images are obtained by short wavelength, the findings seen on the two modalities are different using fluorescein dye. The SW-FAF signal is thought to originate from lipofuscin and melanolipofuscin [9,10,11]. In the damaged RPE cells, lipofuscin or melanolipofuscin can increase or decrease and may show hyperfluorescence or hypofluorescence in SW-AF. In the damaged RPE cells, if atrophic, FA may show a window defect, i.e., hyperfluorescence. If there is an SRD, in FA, hyperfluorescence can be seen, even over the normal RPE, because of pooling. Moreover, the focus can vary on the lesion of leakage of fluorescein dye by using the scanning laser ophthalmoscopy system, which we used in this study.

As the strengths of our research, what we see in NIR-AF and ICGA may not necessarily be the same, but we were able to obtain overlapping findings in many cases. In our previous study, the HFF on late-phase ICGA was thought to correspond to lipid accumulation at Bruch’s membrane to the choriocapillaris level [4], and these HFF were a significant risk factor for an SRD [5]. FA and ICGA testing are laborious and invasive, and SW-FAF is dazzling. However, NIR-AF is the least dazzling and has the shortest image acquisition time than other modalities. NIR-AF may easily detect overlapped findings of RPE disorders and lipids at Bruch’s membrane and may replace the late-ICGA imaging, and it has the possibility to predict the lesion of the occurrence of SRD. Whether the HFF in NIR-AF is a significant risk factor for SRD would be of interest as a future study.

The limitations of this study are as follows. Firstly, we wanted to analyze our data by multiple regression analysis using the data of the duration of subjective symptoms or gender differences. However, it was not possible to determine when the HFF occurred in the contralateral eye. Moreover, we had only two unilateral eyes in women. Therefore, we performed a simple regression analysis instead of multiple regression analysis. The number of patients was too small, and the symptom duration was too broad in this study. We need to obtain follow-up data on many patients in the future study. In addition, we would like to investigate these HFF using deep learning to check the replication in the future study.

## 5. Conclusions

In this study, we could detect any abnormal foci with high probability in SW-FAF, NIR-AF, and FA within the HFF area on late-phase ICGA, and we found NIR-AF easily detected the HFF seen in the late-ICGA visually. FA and ICGA testing are laborious and invasive, but SW-FAF and NIR-AF require short image acquisition time. NIR-AF is the least dazzling and has the shortest image acquisition time than the other modalities, which may easily detect those overlapped findings of RPE disorder and lipids at Bruch’s membrane. It will be interesting to clarify whether the HFF in NIR-AF is a significant risk factor for SRD in the future study. If we prove that, NIR-AF might be able to replace late ICGA images when we evaluate RPE disorders and SRD development.

## Data Availability

The data presented in this study are available on request from the corresponding author.
